# Maintenance of adult stem cells from human minor salivary glands via the Wnt signaling pathway

**DOI:** 10.1186/s13287-023-03445-x

**Published:** 2023-08-25

**Authors:** Bo kyoung Kang, Zhu Zhu, Jian Wang, Jia Zhou, Shun Yu, Xianyu Zhou, Zhenmin Zhao, Aiguo Xie, Lin Lu, Jun Yang

**Affiliations:** 1 Department of Plastic and Reconstructive Surgery, Shanghai Ninth People’s Hospital, Shanghai Jiao Tong University School of Medicine, Shanghai, 200011 China; 2 Shanghai Key Laboratory of Tissue Engineering, Shanghai, 200011 China; 3 Department of Burns and Plastic Surgery, The Affiliated Hospital of Jiangnan University, Wuxi, 214041 China; 4 Department of Plastic Surgery, Peking University 3Rd Hospital, NO.49 of North Huayuan Road, Haidian District, Beijing, 100191 China

**Keywords:** Human minor salivary gland stem cells, Salivary gland regeneration, Stem cell therapy, Organoids

## Abstract

**Background:**

Xerostomia is a salivary gland dysfunction that negatively impacts the life quality of patients; however, there is no effective treatment for xerostomia. Bioengineered organs, generated using stem cells obtained from newborn salivary glands and ligated injury models, are a new organ transplantation strategy that could be feasible for xerostomia treatment. Reconstruction of salivary gland organoids by seed cells obtained from human minor salivary glands will offer theoretical fundaments and technology support for clinical application and organ regeneration research. Herein, we aimed to propose a new method for culturing and enriching adult human minor salivary gland stem cells in vitro in a three-dimensional (3D) environment via Wnt signaling activation.

**Methods:**

Obtained and characterized human minor salivary gland stem cells (hMSGSCs) with self-organization ability were 3D-cultured to generate organoids. We examined hMSGSCs proliferation and colony formation using MTT (3-(4,5-dimethylthiazol-2-yl)-2,5-diphenyltetrazolium bromide) assays. Telomerase reverse transcriptase staining, flow cytometry, immunofluorescence assay, RNA isolation, RT-PCR, and qPCR were performed to assess hMSGSCs structure and the function of reconstructive organoids in vitro.

**Results:**

hMSGSCs showed typical epithelial-like characteristics, such as positive for CD49f and cell KRT expression. hMSGSCs served as adult stem cells in salivary glands and could differentiate into acinar and duct cells. Upon the addition of Noggin, CHIR99021, and Wnt3A to the 3D culture system, hMSGSCs showed higher *LGR5* expression and decreased *AMY1B* and *MUC5B* expression*.* Therefore, the Wnt and bone morphogenetic protein (BMP) pathways are important in regulating hMSGSCs self-organization and differentiation.

**Conclusions:**

We showed that the stem cell properties of hMSGSCs in a 3D culture system can be maintained by activating the Wnt signaling pathway and inhibiting the BMP signaling pathway. Our findings contribute new insights on salivary gland organoid generation in vitro.

**Supplementary Information:**

The online version contains supplementary material available at 10.1186/s13287-023-03445-x.

## Background

Xerostomia is a salivary gland dysfunction caused by various reasons, such as radiotherapy treatment for head and neck tumors [1]. It mainly manifests as decreased salivation and can be accompanied by symptoms, such as dysphagia and inflexible tongue movements, which greatly affect the quality of life of patients. Currently, the treatment for xerostomia is mainly limited to symptomatic treatment to relieve symptoms, and there are no effective treatment methods. With the recent advances in regenerative medicine, cell therapy has provided new ideas for treating xerostomia. In particular, the use of bioengineered organs to replace damaged ones is an attractive organ transplantation strategy.

Salivary glands are important exocrine organs. Okumura [2] and Hisatomi [3] have previously obtained a type of epithelial pluripotent stem/progenitor cells from ligated and damaged submandibular gland tissues in the normal submandibular glands of newborn and adult mice. Kishi et al. [4] used low-density culture methods to obtain single-cell clones of pluripotent salivary gland stem/progenitor cells that simultaneously expressed acinar, duct, and myoepithelial cell markers. They found that the proliferative ability of stem cells in the adult submandibular gland was weaker than that in newborn mice. In 2014, Zhang et al. [5] used in vivo fluorescence tracking to identify the presence of a group of label-retaining cells in the minor salivary glands of the palate, which are located in the lower exocrine ducts, and a minor salivary gland. Some acinar cells can differentiate into KRT5-positive basal cells and KRT8-positive luminal cells in in vivo experiments. However, in human tissues, only reports of epithelial morphological progenitor cells obtained from submandibular and parotid glands are available [6].

Previously, we have epithelial progenitor cells from adult human minor salivary glands and found that they have the potential to differentiate into hepatocytes and can therefore be used to treat liver injury [3]. However, in vitro expansion and purification of human minor salivary gland stem/progenitor cells (hMSGSCs) have yet to be achieved. Yoon et al. [7] refined the culture conditions and successfully established a long-term culture of adult salivary gland organoids stimulated with neurotransmitters. Whether small molecules or growth factors would also enrich epithelial progenitor cells from adult human minor salivary glands remains to be explored. The extracellular matrix plays an important role in tissue generation, organ development, and cell activity. It induces and maintains cell processes, mediates cell adhesion, and provides support for cell structure [8]. Matrigel is a soluble basement membrane extracted from the Engelbreth–Holm–Swarm mouse sarcoma that is rich in extracellular matrix and is very similar in structure to mammalian basement membrane components [9]. In the search for a suitable microenvironment for the survival of adult stem cells, three-dimensional (3D) culture provided by Matrigel was found to have a positive effect. At present, many in vitro experiments involving adult stem cells and related studies have been carried out in the Matrigel-mediated 3D culture system [10, 11].

This study aimed to explore the potential regenerative and therapeutic abilities of human minor salivary gland stem cells maintained in a 3D culture environment upon the activation of the Wnt signaling pathway. Our findings contribute new insights on salivary gland organoid generation in vitro and pave the development of treatment strategies for xerostomia.

## Methods

### Isolation and culture of hMSGSCs

Human minor salivary glands were obtained from mucosal tissues discarded during reconstructive surgeries for cleft lip patients. The gland tissues were minced into 0.4–0.5-mm^3^ pieces, washed with Dulbecco’s phosphate-buffered saline (PBS; Sigma-Aldrich, MO, USA), and placed on the bottom of the culture flask. The flasks were incubated for 4–5 h at 37 °C and 5% CO_2_, and 5 mL of culture media was added to the flasks. Keratinocyte medium (Sciencell Research Laboratories, CA, USA), containing 5 μg/mL bovine serum albumin, 50 μg/mL bovine pituitary extract, 5 μg/mL transferrin, 3.75 μg/mL insulin, 3 ng/mL fibroblast growth factor 2, 1 ng/mL epidermal growth factor, 500 ng/mL epinephrine, 0.5 μg/mL hydrocortisone, 10^–8^ M prostaglandin E_2_, and 30 nM T_3_. hMSGSCs were isolated by clone ring and cultured according to the described in our previous study [12]. The cultured cells were passaged using 0.25% trypsin (HyClone, USA) at 85% confluence in a 1:3 split ratio.

### 3D culture of hMSGSCs in vitro

A total of 500 μL of Matrigel Growth Factor Reduced (GFR) Basement Membrane Matrix (BD #356,230, USA) were used in this experiment and thawed on ice until it was completely dissolved. A 24-well plate was placed in a 37 °C incubator, and the experiment tip was precooled at 4 °C. Upon reaching 85% confluency, the cultured cells were trypsinized to obtain a single-cell suspension. The fully dissolved Matrigel was mixed with an equal volume of keratinocyte medium, diluted into a centrifuge tube moistened with the complete medium, and placed on ice before use. The cells were inoculated in a 24-well plate at a density of 5 × 10^3^ cells per well, after which the counted cell suspension was aspirated and centrifuged, discarding the supernatant. The diluted Matrigel (30–50 μL) was added to the cells, and the 24-well plate was placed in a 37 °C incubator for 20 min to fully solidify Matrigel. Then, 500 μL of the medium was added to each well. The medium was changed every other day. To passage the cell spheres cultured in 3D method, the wells were washed with PBS twice, and 500 μL of 0.25% trypsin (Hyclone, USA) was added to each well. The Matrigel was carefully separated with a 1-mL pipette tip, and the coagulated Matrigel was mechanically dispersed. The plate was then placed in a 37 °C incubator for 5 min. After incubation, the single-cell suspension in the culture well was transferred to a centrifuge tube, and digestion was stopped using a complete medium. Centrifugation was performed at 233 g (times gravity) for 5 min, after which the supernatant was discarded, and the pellet was resuspended in a complete medium and centrifuged again at 1 000 rpm for 5 min. The method described above was used to inoculate a single-cell suspension in Matrigel, a ratio of 1:6 hMSGSCs was used in a new 3D system. To activate different signaling pathways, different drugs were added on day 4. The concentration of drugs used in the media was described as follows. Wnt 3A group contained 100 ng/mL Wnt 3A (PeproTech), Noggin group was supplemented with 100 ng/mL Noggin (PeproTech), CHIR99021 group added 1 μM CHIR99021(Biogems, Colinas, CA, USA). The medium was changed every other day. The diameter of each cell spheres was quantified using ImageJ, in Additional file 2: Fig. S1.

### Human telomerase reverse transcriptase (hTERT) staining and immunofluorescence assay

The cells were grown to 70% confluency and prepared for cell immunofluorescence detection. The cells were fixed with ice acetone and methanol at a volume ratio of 1:1 at –20 °C for 10 min, after which the fixative was discarded, and 1 mL 2 N hydrochloric acid was added to each compartment and incubated for 20 min. Hydrochloric acid was discarded and neutralized with 1 mL of 0.1 M sodium borate solution via incubation for 5 min. The sodium borate solution was discarded, and the blocking solution was added and incubated at 27 °C for 2 h. The blocking solution was discarded, and the diluted hTERT primary antibody (Additional file 1: Table S1) was added and incubated at 4 °C overnight. The next day, the cells were washed with PBS four times for 5 min each at room temperature. The secondary antibody was added and incubated for 2 h at room temperature, followed by washing with PBS four times for 5 min and adding 4′,6-diamidino-2-phenylindole (DAPI). For the immunofluorescence staining assay, the sections or cells were permeabilized with 0.2% Triton X-100 (Sigma, USA) for 10 min and blocked in 10% fetal bovine serum (Gibico, USA) at room temperature for 1 h. They were incubated with primary antibodies at 4 °C overnight and washed with PBS supplemented with 1% bovine serum albumin, followed by incubation with secondary antibodies at 27 °C for 30 min on the next day. The cells were mounted with DAPI and imaged using a Nikon TE2000-S microscope (Nikon, Tokyo, Japan). The antibodies used are listed in Additional file 1: Table S1.

**Table 1:** Flow cytometry analysis of human minor salivary gland stem/progenitor cells

Markers	Average	SD
CD29	99.83%	0.27%
CD49f	99.50%	0.80%
CK-KIT	99.25%	0.35%
HLA-ABC	99.15%	0.56%
HLA-DR	0.23%	0.04%
CD117	–	–
SSEA-1	3.78%	0.49%
CD90	2.67%	0.05%

The averaged percentage values of cells positive for the cell markers are shown. Each marker was tested for passage 3 cells derived from three different samples. SD: standard deviation

### Cell proliferation and colony formation assays

At passages 5 and 15, hMSGSCs were cultured in 96-well cell plates at a density of 1 × 10^4^ cells. Growth curves were drawn, and cell doubling time was calculated via the MTT assay (Sigma-Aldrich, MO, USA). Crystal violet was used to stain the cells for the colony formation assays [13]. The cell population doubling time was estimated as described [14], Patterson formula: Td = T × lg2/(lgN2—lgN1), Td is the doubling time (in hours), N is the number of cells and T is the time for cell growth from N1 to N2.

### Flow cytometry analysis

hMSGSCs cultures at passage 3 were harvested from 60 mm dishes at 80% confluency for flow cytometry. A total of 1 × 10^6^ cells/mL were used for each test. For cell surface marker analysis and intracellular antibody staining, the sample cells were washed, fixed, and incubated with antibodies, as previously study described [12]. This study used FACS AriaTM II (BD Biosciences, NJ, USA) for the analyses. FlowJo version 7.6.1 (Tree Star Inc., OR, USA) was used to analyze the acquired data. The antibodies used are listed in Additional file 1: Table S1.

### RNA extraction, cDNA synthesis, real-time quantitative polymerase chain reaction

Total RNA was using the RNeasy Mini kit (Qiagen, Germany) according to the manufacturer’s instructions and was quantified using a NanoDrop 2000c instrument (Thermo Scientific, USA). RNA was reverse transcribed using M-MLV (Invitrogen) according to the manufacturer’s instructions. Real-time quantitative polymerase chain reaction was performed on a Light-Cycler Roche480 (Roche Molecular Systems, Pleasanton, CA, USA). Glyceraldehyde 3-phosphate dehydrogenase was used as the endogenous control. The primers are listed in Additional file 1: Table S2.

### Statistical analysis

Statistical analysis was performed by two-tailed paired t-test or by one-way ANOVA test to using the SPSS 22.0 and GraphPad Prism 9 (GraphPad Software Inc., CA). Data are presented as the mean ± standard deviation (SD), *P-values* < 0.05 were considered statistical significance.

## Results

### hMSGSCs show enhanced self-renewal ability in vitro

Human minor salivary gland tissues were cultured in a keratinocyte medium using the tissue adherence method [12]. Cell colony formation could be observed around the adherent tissue explant on day 7, and the cells showed homogeneous keratinocyte-like morphology. Migrated cells showed strong proliferative ability during passaging and expansion. With reasonable control of cell growth and seeding density, the cells can be passaged every 3–4 days to maintain high proliferation efficiency and are herein termed hMSGSCs.

**Fig. 1:**
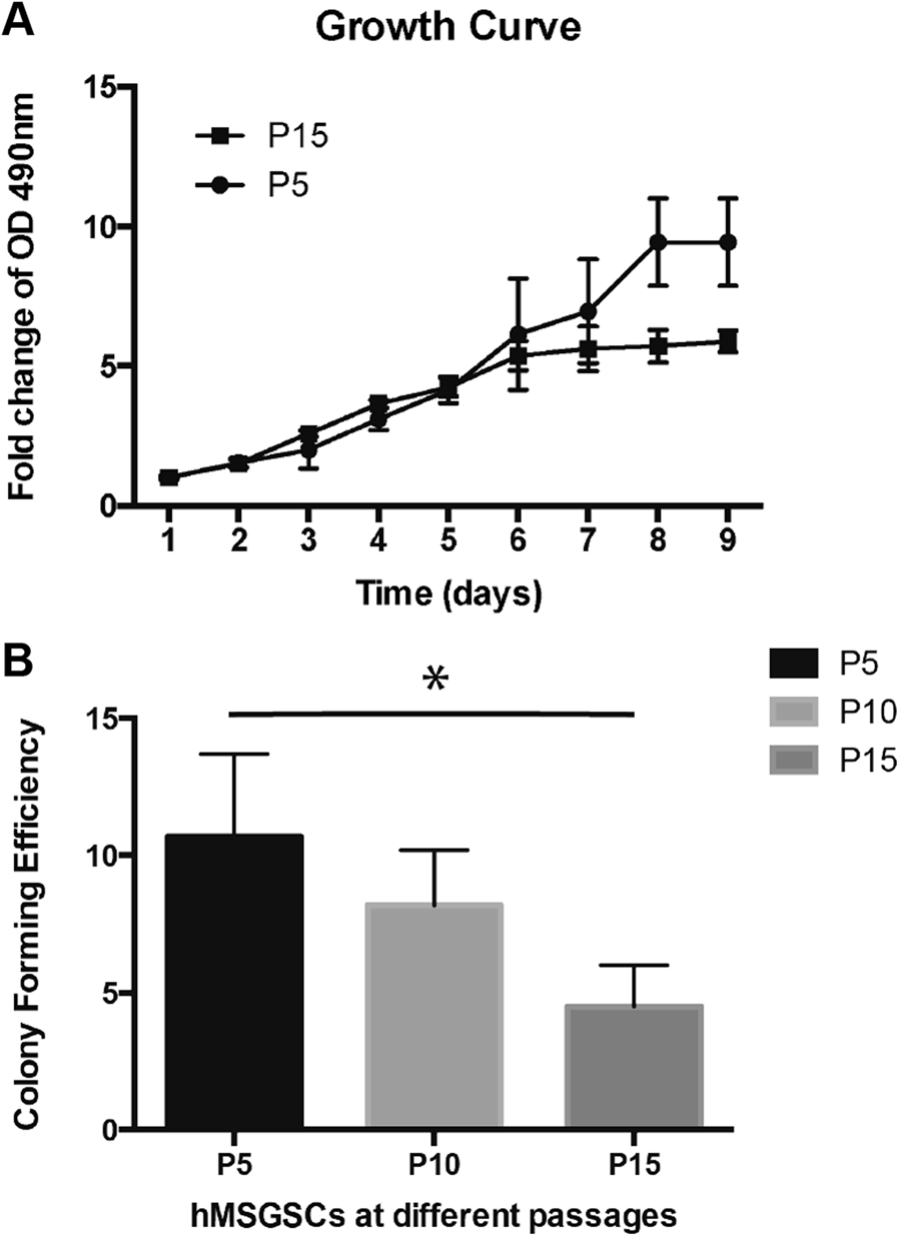
Assessment of human minor salivary gland stem/progenitor cells. **a** Cell growth curve of hMSGSCs for passages 5 and 15. **b** Assessment of hMSGSC colony forming efficiency at passages 5, 10, and 15 (n ≥ 3). *P < 0.05

To clarify their self-renewal ability and determine the influence of subculture on this self-renewal ability, we detected the proliferative ability of cells at passages 5 and 15 (Fig. 1A). The population doubling time of the cells at passages 5 and 15 was 39.71 ± 1.94 h and 39.8 ± 6.99 h, respectively, and was not significantly different.

To further verify the self-renewal ability of the epithelial stem cells of the minor salivary glands, hTERT was tested. The positive expression of hTERT in the nucleus was clearly observed (Fig. 3).

We examined the colony formation ability of hMSGSCs at different generations. Giemsa staining showed that the clone formation rate of cells at passages 5, 10, and 15 was 10.7% ± 3.0%, 8.0% ± 2.0%, and 4.5% ± 1.5%, respectively. The cell doubling time did not change significantly, but the colony formation ability gradually decreased with in vitro passaging (Fig. 1B).

### hMSGSCs show epithelial characteristics

Flow cytometry was performed on hMSGSCs cultured to passage 3 (Table 1), and cell adhesion-related molecules CD29 and CD49f were positively expressed in the cells, which showed epithelial characteristics. Flow cytometry analysis results showed that hMSGSCs did not express the hematopoietic stem cell marker CD117 and rarely expressed the mesenchymal stem cell marker CD90.

**Fig. 2:**
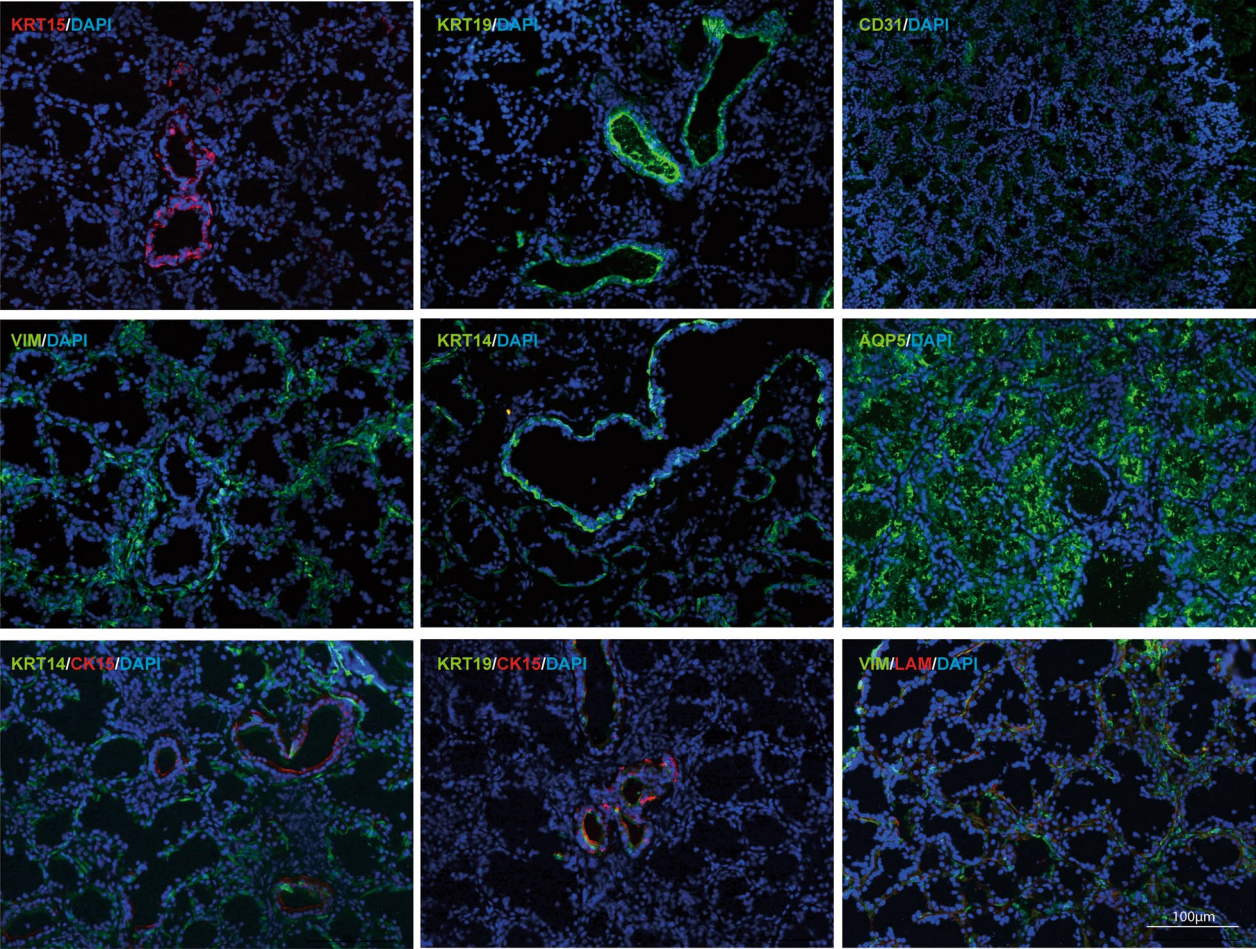
Immunofluorescence analysis of human minor salivary gland tissues. Human minor salivary gland tissues were detected for the expression of KRT15, LAM (red), KRT19, CD31, VIM, KRT14, AQP5 (green). Nuclei were counterstained with DAPI (blue). Scale bar, 100 μm

**Fig. 3:**
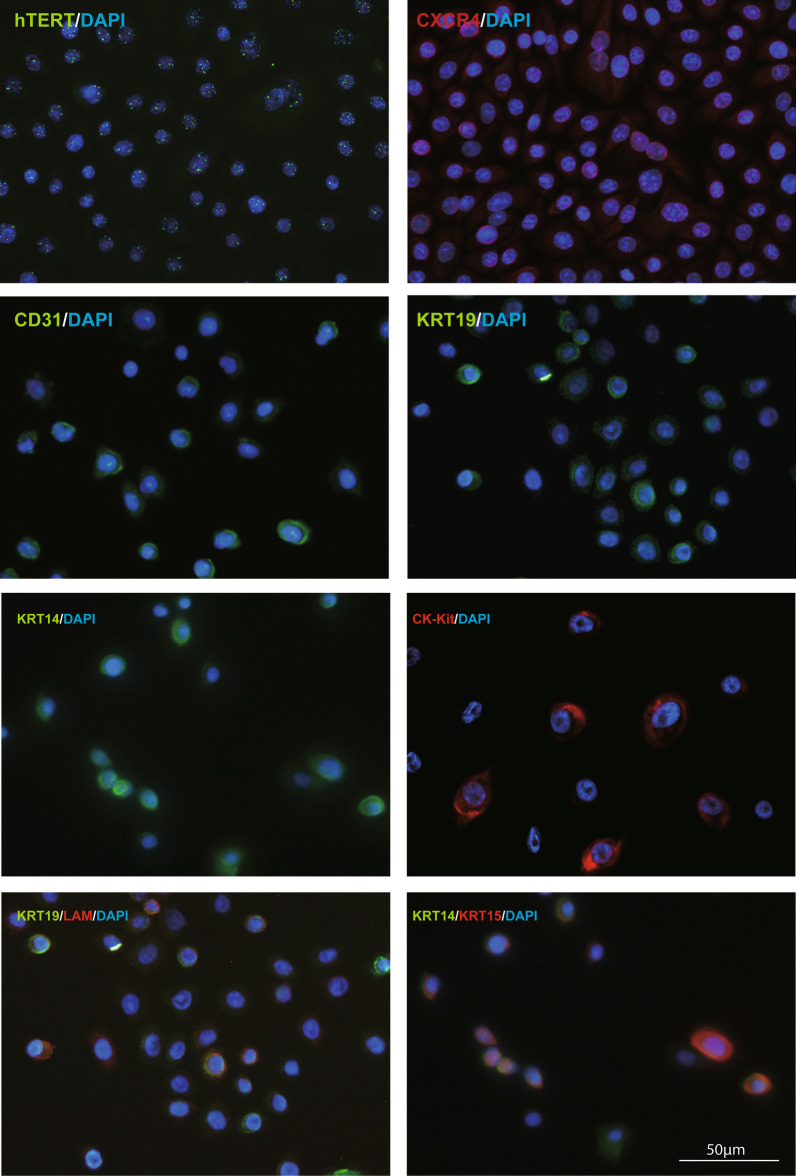
Immunofluorescence assay of hMSGSCs cultured in vitro in a 2D system. The expression of CXCR4, CK-kit, LAM, KRT15 (red), hTERT, CD31, KRT14, and KRT19 (green) were detected. Nuclei were counterstained with DAPI (blue). Scale bar, 50 μm

To elucidate the characteristics of the cells and their origins, we carried out different types of cytokeratin detection on minor salivary gland tissues and hMSGSCs cultured in vitro (Fig. 2,3). To determine the source of these cell groups, keratin (KRT) expression was detected in the minor salivary glands. Epithelial progenitor marker KRT15 and KRT19 were expressed in the ducts of the glands, whereas KRT14 was expressed at the base of most ducts and acini. Aquaporin 5 (AQP5) was specifically expressed in the acinus, whereas the interstitial-specific protein vimentin was only expressed in the interstitial part of the glands, and the laminin expressed in the basal part overlapped with vimentin (Fig. 2). Most hMSGSCs positively expressed KRT 14, KRT19, and KRT15. When the passaged cells were co-stained with KRT14 and KRT15, KRT15-negative cells were detected, suggesting that the cells cultured in vitro may be mixed with different types of cell populations (Fig. 3).

### hMSGSCs show spontaneous glandular differentiation potential when cultured in 2D platform

**Fig. 4:**
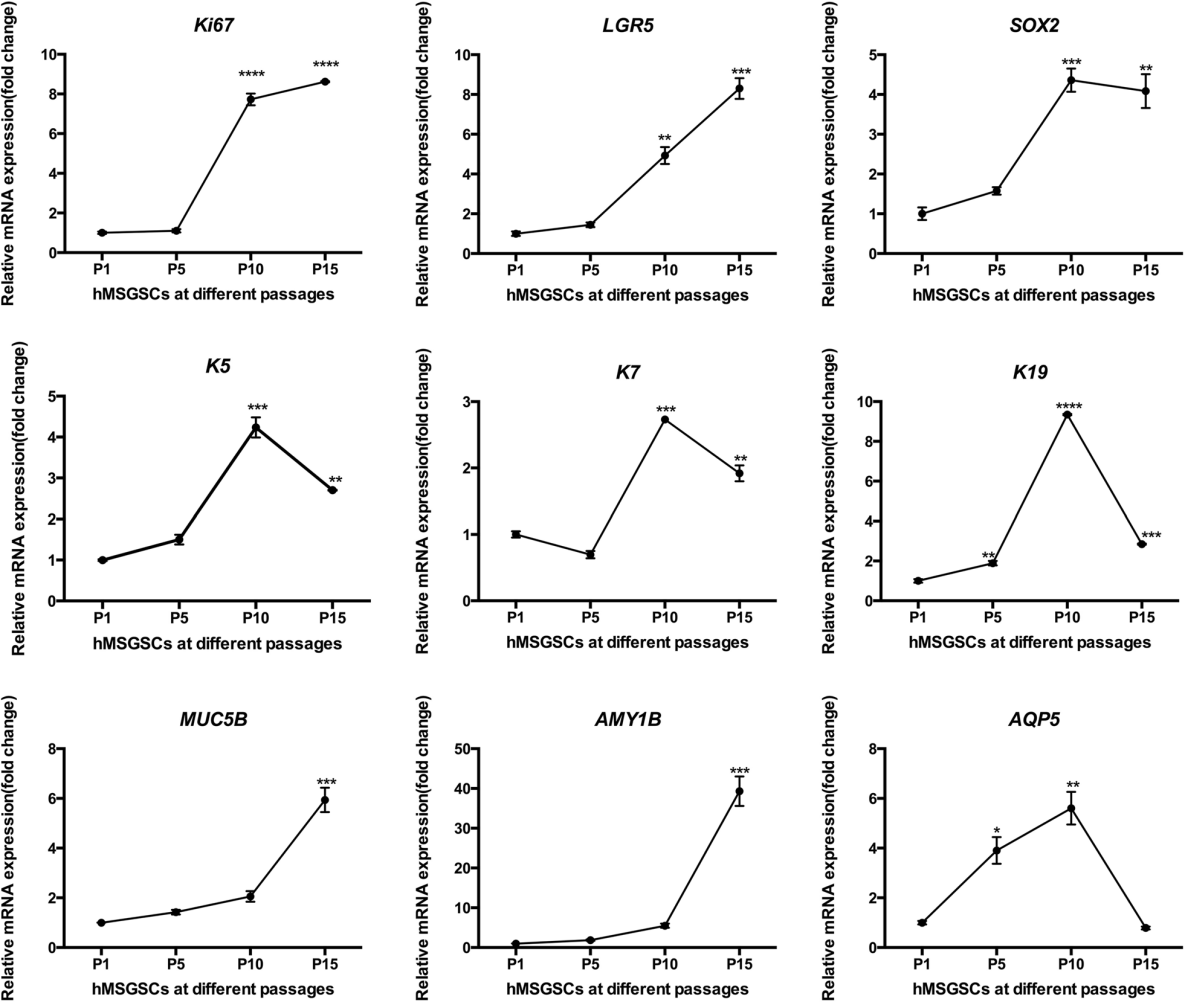
Gene expression of different passages of hMSGSCs in vitro. Quantitative real-time PCR analysis of marker (Ki 67, LGR5, SOX2, K5, K7, K19, MUC5B, AMY1B, and AQP5) expression on hMSGSCs under different passages (n ≥ 3). *P < 0.05, **P < 0.01

hMSGSCs cultured to passages 1, 5, 10, and 15 on two-dimensional (2D) plates were collected for the detection of gene expression (Fig. 4). We separately determined the expression of genes related to stem cell characteristics, epithelial cell-related KRT, and salivary acinar glands. With hMSGSCs passaging in vitro, the expression of the cell proliferation marker Ki67 continued to increase. Compared with passage 1, the increase was most evident between cells at passages 5 and 10 (*P* < 0.001). The adult stem cell marker leucine-rich repeat-containing G-protein coupled receptor 5 (LGR5) also showed a similar trend, gradually increasing during cell culture in vitro. The expression of another embryonic stem cell-related marker, SRY-box 2 (SOX2), increased initially and then decreased, reaching the highest at passage 10. Similarly, the expression of the epithelial cell-related proteins KRT5, KRT7, and KRT19 increased at first and then decreased, reaching the highest at passage 10. We also detected epithelial markers associated with salivary glands, including mucin 5B (MUC5B), amylase alpha 1B (AMY1B), and AQP5. The expression of these genes related to the cell differentiation increased, with that of AQP5 reaching the maximum at passage 10.

### hMSGSCs maintain self-renewal ability in a 3D culture system

**Fig. 5:**
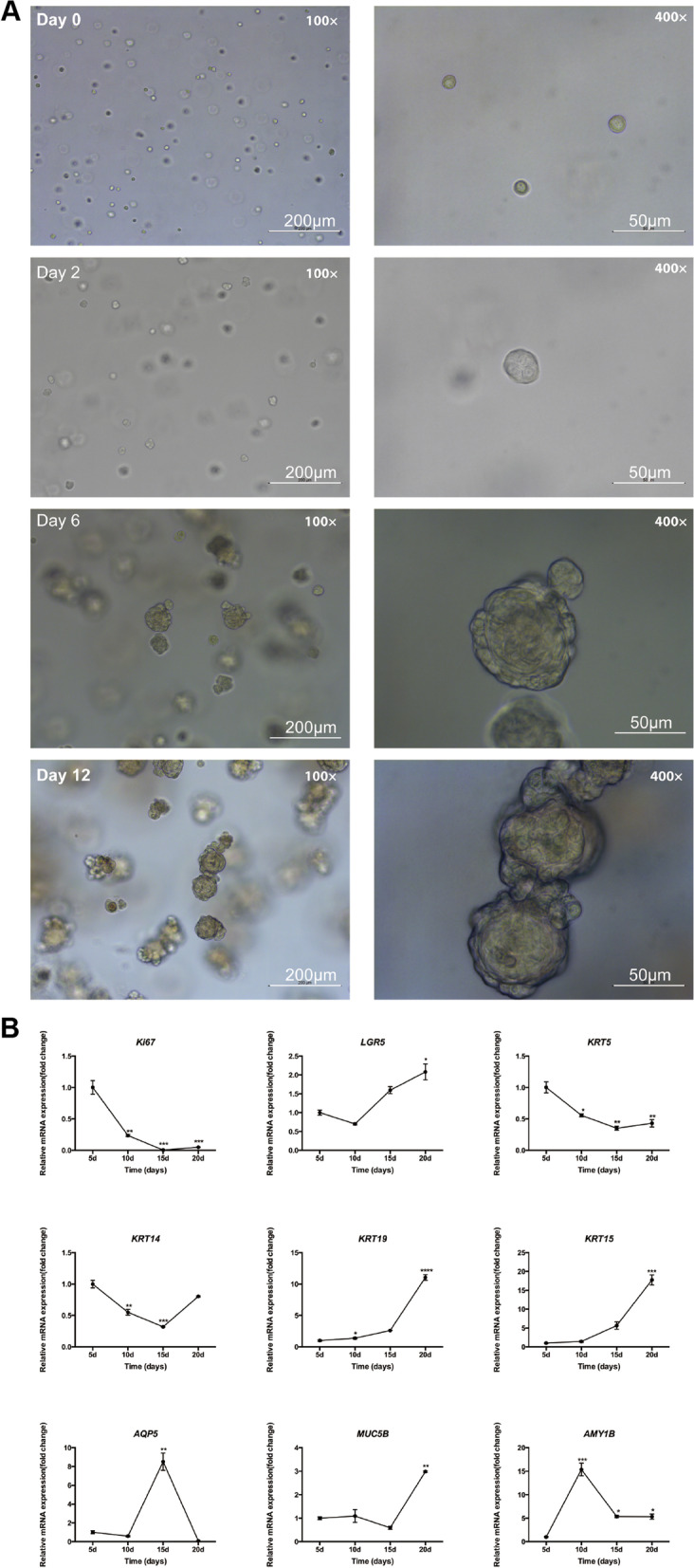
hMSGSCs cultured in a Matrigel 3D system. **a** Sphere formation of hMSGSCs cultured in a Matrigel 3D system. Bright field microscopy image of self-organized hMSGSCs in Matrigel. Representative image at four different culture time (day 0 [day before start of culture], 2, 6, and 12). Scale bar, 200 μm (left) and 50μm (right). **b** Gene expression of hMSGSCs cultured in a 3D system. Quantitative real-time PCR analysis of the relative mRNA expression of various markers (Ki67, LGR5, KRT5, KRT14, KRT19, KRT15, AQP5, MUC5B, AMY1B) at different times (day 5, 10, 15, and 20) (n ≥ 3). *P < 0.05, **P < 0.01

The hMSGSCs were inoculated into Matrigel (Fig. 5A), and cell sphere formation was observed after 2 days. At day 6, cell spheres began to form, with a diameter of approximately 50 μm. The surface of the spheres was not smooth and had a high refractive index. We observed cell spheres growing with a diameter ranging from 50 to 100 μm until day 12. When the culture was continued, a black substance appeared in the center of the cell pellets, indicating cell apoptosis. The cell spheres were digested with trypsin to form a single-cell suspension and inoculated into new Matrigel. After 6 days, the formation of cell clones was observed under the microscope, suggesting that the 3D culture can maintain the self-renewal ability of salivary gland stem cells in the cell population. Thus, hMSGSCs in a 3D culture system was confirmed to be passaged stably.

To identify the status of cells cultured in a 3D system, we collected spheres at days 5, 10, 15, and 20 for gene expression detection (Fig. 5B). As the duration of in vitro culture increased, the expression of Ki67 decreased, whereas that of LGR5 increased, with a significant difference between cells harvested on different days. In addition, the expression of the stem/progenitor cell marker KRT5 decreased, whereas that of KRT15 and KRT19 continued to increase during culture. Regarding the mature acinar cell markers AQP5, MUC5B, and AMY1B, AQP5 expression in the 3D system increased, whereas that of MUC5B and AMY1B decreased during the first 10–15 days. The expression of MUC5B increased, whereas that of AQP5 and AMY1B declined from day 15, possibly due to apoptosis and lack of self-renewability.

**Fig. 6:**
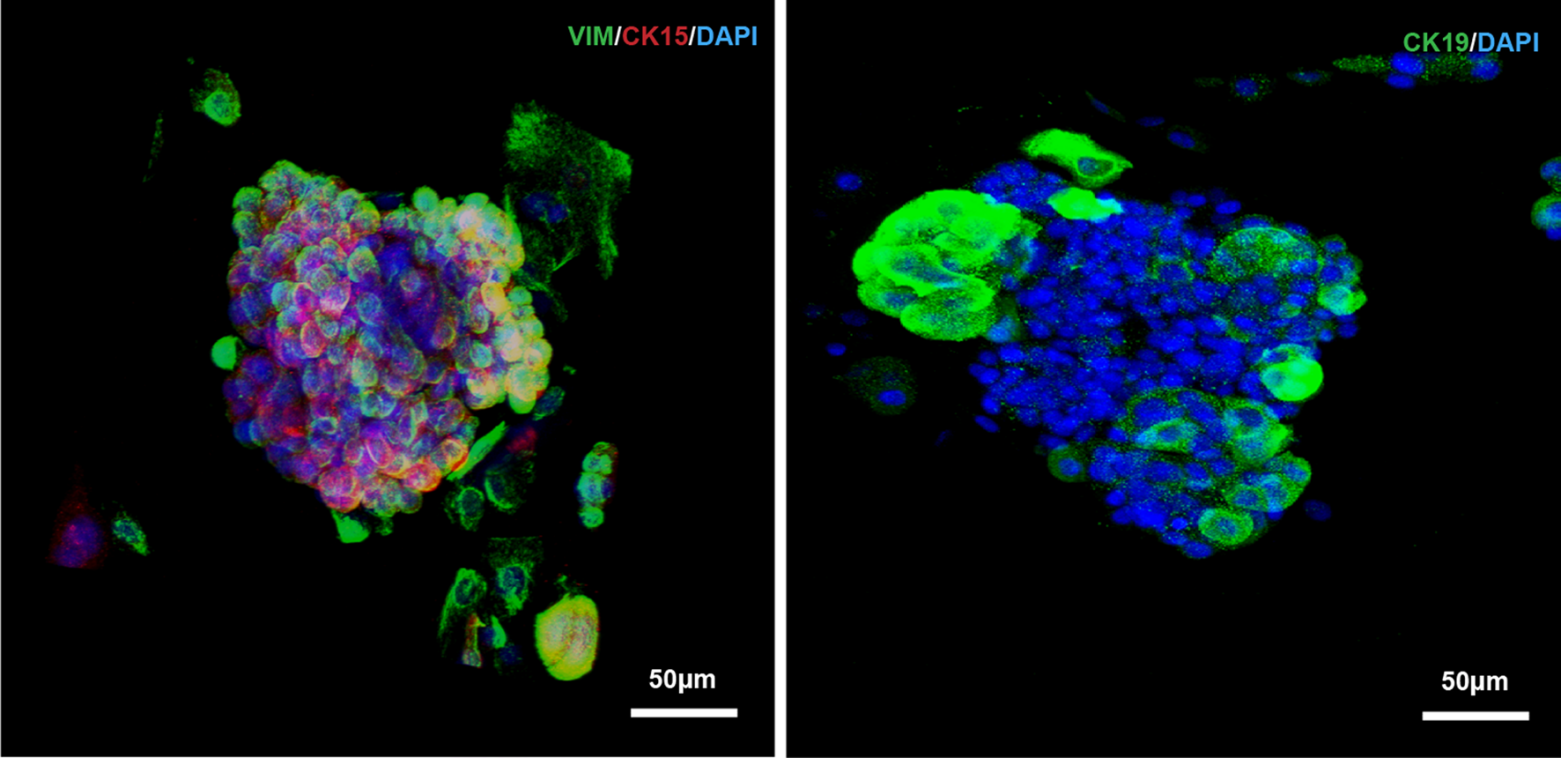
Immunofluorescence assay of hMSGSCs cultured in 3D system. The expression of CK15 (red), VIM, CK19 (green) were detected. Nuclei were counterstained with DAPI (blue). Scale bar, 50 μm

To further confirm hMSGSCs cell status in 3D culture system, we performed immunofluorescence test on spheroids in vitro*.* VIM and cytokeratin 15(CK15) positively expressed in spheroids, while CK19 scattered expressed in hMSGSCs upon periphery part of speroids (Fig. 6).

### Cell spheres maintain stem cell properties upon Wnt signaling pathway activation

**Fig. 7:**
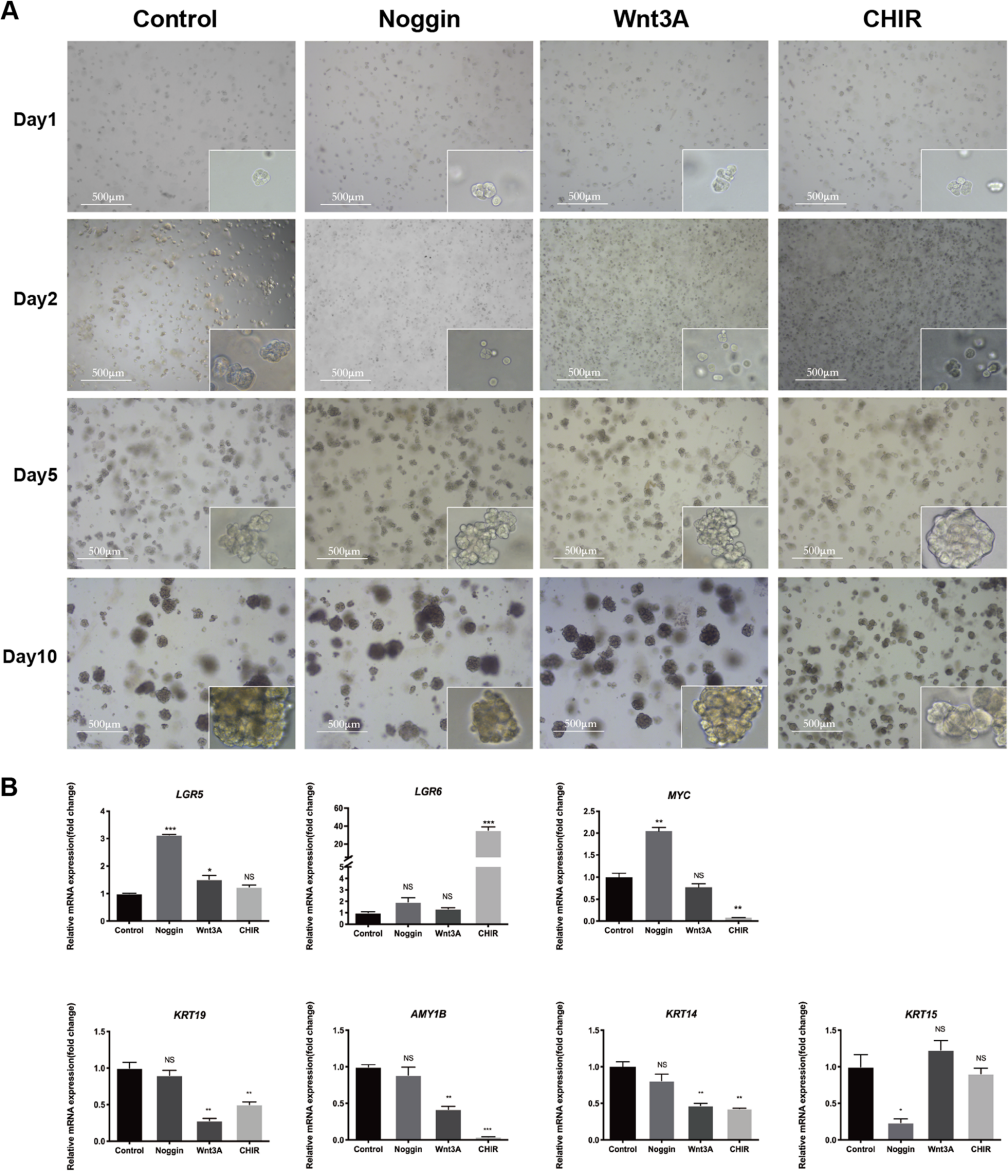
hMSGSCs growth in Matrigel upon supplementation with different signal pathway factors. **a** hMSGSCs cultured with Noggin, Wnt3A, and CHIR99021. Scale bars, 500 μm. **b**. Gene expression of hMSGSCs cultured with Noggin, Wnt3A, and CHIR99021 (n ≥ 3). *P < 0.05, **P < 0.01

Since hMSGSCs had better cell renewability in the 3D culture system, we further explored the optimal microenvironment to maintain and purify them in cell spheres. Pharmacological activation of Wnt and bone morphogenetic protein (BMP) signaling pathways was achieved by adding the small molecules Noggin, Wnt3A, and CHIR99021, and spheres were cultured in a 3D platform for 10 days (Fig. 7A). All four groups (control, Noggin, Wnt3A, and CHIR99021) were passaged onto Matrigel-coated wells at the same cell density at day 0 (day before start of culture), and uniform inoculation was observed at day 1. With time, the spheres size continued to grow in the groups, except for the CHIR99021 group, which showed a much more uniform and smaller sphere size and significantly reduced cell apoptosis at day 10, and more spheres were dissociated in the control group than in the other groups. (Control group cell spheres diameter was 127.45 ± 5.72 μm, Noggin group spheres diameter was 196.26 ± 20.91 μm, Wnt3A group spheres diameter was 183.86 ± 14.23 μm and CHIR99021 spheres diameter was 62.76 ± 10.81μm) (Additional file 2: Fig. S1).

Wnt3A and CHIR99021 (GSK3 inhibitor) were added to the culture system to activate the canonical Wnt signaling pathway. Compared with the Noggin and CHIR99021 groups, the stem cell-related marker LGR6 had the highest expression level in the CHIR99021 group. However, the expression of the glandular duct and acinar-related markers KRT19, KRT14, and AMY1B was significantly reduced in the Wnt3A and CHIR99021 groups. The BMP antagonist Noggin was also added to the culture system. The expression of the stem cell-related marker LGR5 was higher in the Noggin group than in the other groups. The Noggin group also showed significantly higher expression of MYC, another stem cell-related marker. KRT15 had the lowest expression level in the Noggin group, indicating inhibition of cell differentiation. Meanwhile, the expression of KRT19, KRT14, and AMY1B in the Noggin group was significantly higher than that in the Wnt group (Fig. 7B). However, it was not significantly different compared with that in the control group. These results indicate that the Wnt signaling pathway plays an important role in maintaining the stem cell properties of hMSGSCs.

## Discussion

In this study, we obtained stem/progenitor cells from adult human minor salivary gland tissues via tissue culture and cultured these hMSGSCs in vitro until passage 25. Analysis of the proliferation curve and cell clone formation rate revealed that the hMSGSCs have high self-renewal capacity. We also compared the growth curve and cell clone formation of hMSGSCs at passages 5 and 15 and found that in the latter, the subculture time increased, whereas the clone formation rate declined, indicating that the cell population that formed in the primary culture was a mix of cell types, including epithelial stem cells, progenitor cells, and certain acinar and ductal cells [15, 16]. Only epithelial-derived stem cells retained the potential to form clones after passage 15, whereas the other types of cells may cause apoptosis or develop into end-stage cells.

The hMSGSCs exhibited significant epithelial cell characteristics, strongly expressing KRT14, KRT15, and KRT19, integrins CD29 and CD49f, and laminin. This is consistent with the characteristics of epithelial progenitor cells obtained from the submandibular gland [6]. However, unlike the epithelial progenitor cells obtained in a mouse ligation model, hMSGSCs did not express c-Kit. Sato et al. [6] also reported that labeling cells with c-Kit did not result in more salivary gland progenitor cells in the flow-sorting process, indicating that c-Kit is not a marker of salivary gland stem/progenitor cells in normal tissues.

After performing co-staining for KRT14, KRT15, and KRT19 in pairs, we found that their expression in hMSGSCs differed and that KRT19 and KRT15 were not completely expressed in all stem/progenitor cells. KRT is the main component of the cytoskeleton and functions to maintain cell shape, complete cell movement, and resist external mechanical stress. There are more than 20 different subtypes of the KRT family, the expression of which is tissue specific. The expression of KRT differs according to the cell type, embryonic development stage, cell growth, microenvironment, and degree of cell differentiation. Generally, the monolayer epithelium expresses KRT7, KRT8, KRT18, and KRT19, whereas the stratified epithelium expresses KRT5, KRT14, and KRT15.

In primary mammary progenitor cells arise during embryogenesis, KRT14 is expressed in the suprabasal and luminal compartments, as reported by Sun et al. [17]. KRT19 and KRT14 double-positive epithelial cells could differentiate into ductal cells and muscles [18]. Based on the immunofluorescence staining results of the minor salivary gland tissue and stem/progenitor cells, the obtained minor salivary gland stem/progenitor cells are suggested to originate from the base of the duct basal of tissue. In the minor salivary gland, KRT19 and KRT15 are expressed in epithelial cells from duct parts. Our results showed that in the in vitro culture, the characteristics and markers of hMSGSCs varied during passaging, indicating spontaneous differentiation.

**Fig. 8:**
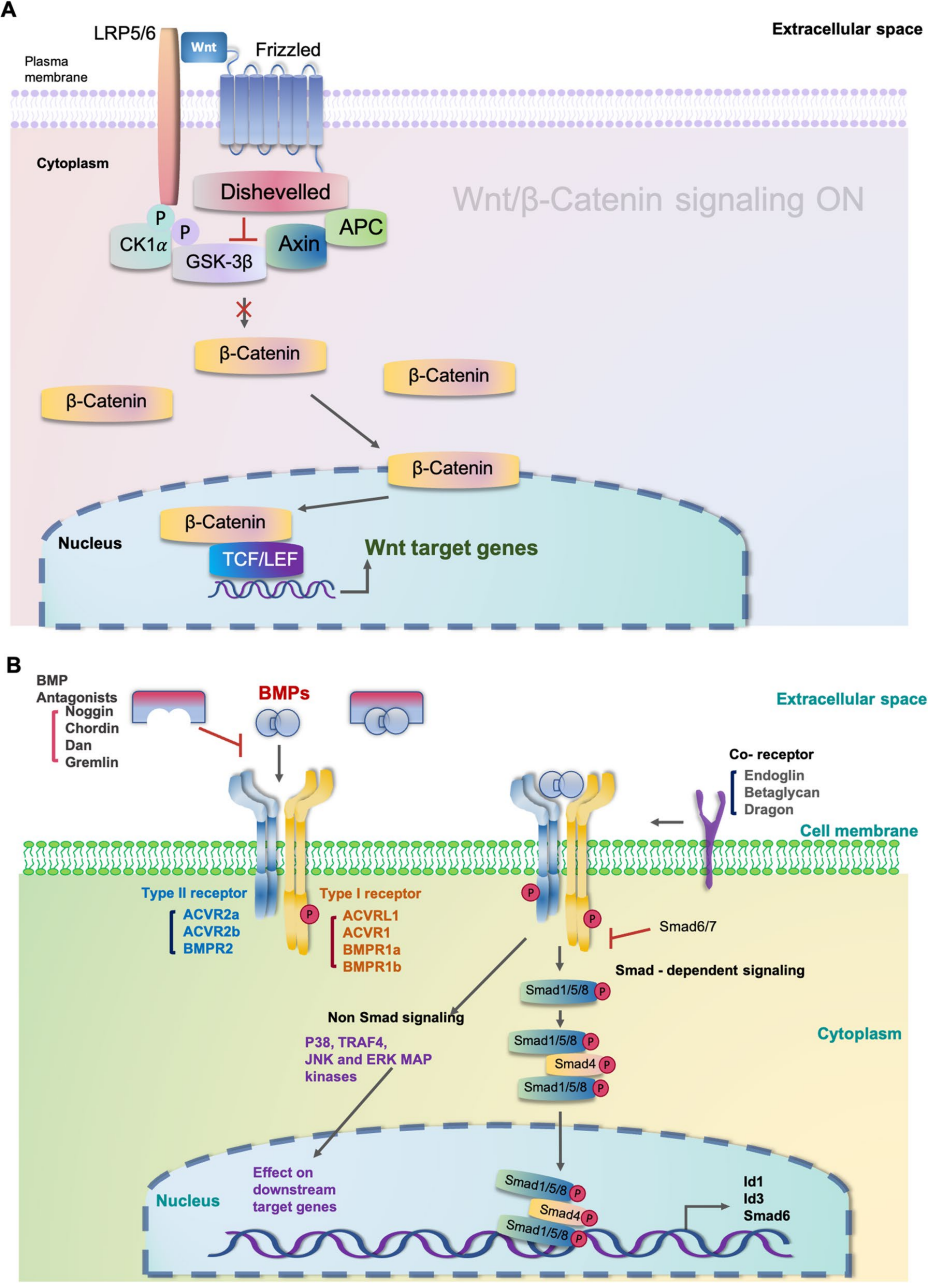
Schematic images of canonical Wnt/β-catenin and BMP signaling pathways. **a** Canonical Wnt/β-catenin signalling. The Wnt signaling pathway has two types: canonical (dependent Wnt/β-catenin signaling pathway) and noncanonical (independent Wnt/β-catenin signaling pathway) Wnt signaling pathways. When a Wnt ligand binds to the Frizzled receptor and LRP5/6, the Wnt/β-catenin pathway is activated. Glycogen synthesis kinase 3β (GSK3β) is the promoter of the Wnt/β-catenin pathway. When GSK3β activity is inhibited by drugs, β-catenin is translocated into the nucleus, activating the Wnt pathway. **b** BMP signalling. BMPs signals are transmitted by type I and II receptor complexes. BMPR activation can cause non-Smad signaling by activating the p38, JNK, and ERK MAP kinases. Noggin is a BMP antagonist that can reduce BMP pathway expression

*LGR5* and *LGR6*, categorized as R-spondin receptors and belonging to the *LGR* superfamily, are excellent markers for self-renewal and differentiation of stem cells in the epithelia, such as the intestine and skin [19], and potentiates Wnt signaling [20, 21] (Fig. 8a). In hMSGSCs, the expression of the stem cell-related markers LGR5 and SOX2 increased after passaging in vitro. Similarly, the expression of Ki67 was increased which represents cell renewal, thus suggesting that minor salivary gland stem cells were enriched during culture. In contrast, the expression of KRT5 and KRT19 continued to increase, reaching a peak, and then decreased. The *MUC5B* and *AMY1B* genes encode mucin and salivary amylases, respectively, which are commonly expressed in acinar epithelial cells. During in vitro culture, their expression increased, suggesting that some stem/progenitor cells have spontaneously differentiated into acinar epithelial cells during passaging.

When hMSGSCs were cultured in a 3D Matrigel system, the expression of the stem cell marker LGR5 increased, whereas that of the mature acinar cell marker AMY1B increased first and stabilized at a certain level. This change in the gene expression profile was similar to that of the cells at passage 20 cultured in a 2D system; however, the time was significantly shortened, and the expression of stem cell markers was increased. This result is consistent with a previous finding reporting that 3D culture is beneficial for the maintenance of stem cell characteristics [11], suggesting that our proposed 3D culture method is more suitable for in vitro culture and enrichment of hMSGSCs than the 2D culture method.

The Wnt signaling pathway is a highly conserved pathway that participates in the regulation of cell proliferation, migration, and differentiation [20]. It can be classified into two types: canonical (dependent Wnt/β-catenin signaling pathway) and noncanonical (independent Wnt/β-catenin signaling pathway) Wnt signaling pathway [22–24]. The Wnt/β-catenin signaling pathway plays an important regulatory function in the postnatal growth and regeneration of the mouse submandibular gland [24]. According to Hai et al. [20], inhibition of the β-catenin signaling pathway in the epithelium altered the development of the submandibular gland after birth. In addition, its stimulation in the epithelium enhanced ductal cell proliferation and increased the fraction of salivary gland progenitor cells. Furthermore, Miletich et al. [26] found that the canonical Wnt signaling pathway suppressed the formation of salivary gland epithelial branches, whereas the noncanonical route promoted duct maturation.

In this study, we used Wnt3A to activate the canonical Wnt signaling pathway and found an increased expression of LGR5, indicating that the stem cell status was maintained, and the hMSGSCs were enriched in this manner. According to Suzuki et al. [21], in SMG tissues cultured at the bud stage, constitutive activation of the canonical Wnt/β-catenin signaling inhibited branching morphogenesis, whereas its inhibition accelerated branching morphogenesis. Furthermore, Wnt3A (canonical Wnt/β-catenin signaling ligand) inhibited acinar cell development while maintaining progenitor cell status, indicating that canonical Wnt/β-catenin signaling regulates end bud differentiation [27].

When a Wnt ligand binds to the Frizzled receptor and the LRP5/6, which is a low-density lipoprotein receptor related protein or with its close relative LRP5, Wnt/β-catenin pathway is actuated [28, 29]. The emergence of the Wnt–Fz–LRP6 complex along with the LRP5/6 phosphorylation, and Axin complex are attracted to the receptors [28, 29]. As a consequence, inhibition of Axin-mediated β-catenin phosphorylation takes place, leading to the stabilization of β-catenin, which accumulates and proceeds to the nucleus to institute complexes with TCF/LEF, thereby activating Wnt target gene expression [29] (Fig. 8a).

The CHIR99021 used in this study is an inhibitor of GSK3β. Glycogen synthesis kinase 3β (GSK3β) is considered the promoter of the Wnt/β-catenin pathway [23]. When GSK3β activity was inhibited by drugs, β-catenin translocated into the nucleus and activated the Wnt pathway [20] (Fig. 8a). After activating the canonical Wnt signaling pathway using a recombinant Wnt3A protein and CHIR99021, the expression of LGR5 and LGR6 in hMSGSCs increased, whereas that of KRT19 in ductal and acinar cells and that of AMY1B was inhibited, indicating stem/progenitor cell enrichment and inhibition of duct and acinar differentiation, respectively [28]. Interestingly, we also found decreased KRT14 expression, suggesting that this cell marker may also be related to duct branch formation.

BMP is a secreted growth factor belonging to the transforming growth factor beta superfamily [30, 31]. During embryonic development, the BMP protein plays an important role in the fate selection of epithelial progenitor cells [31]. Noggin, identified in *Xenopus laevis* as a dorsalizing agent and has been detected in various tissues [32, 33], is a BMP antagonist that can reduce BMP pathway expression [34–37]. Interaction inhibited BMPs from binding to their cell surface receptors, limiting BMP signaling in target cells and regulating BMP activity in several body regions [33, 38–40] (Fig. 8b). The interaction of BMP and Noggin is crucial for proper embryonic development [33, 39, 40].

In this study, we found that adding Noggin to the culture increased the expression of stem cell-related genes *LGR5* and *MYC* in hMSGSCs, whereas decreasing the expression of *KRT15* in ductal cells. These findings suggest that after the inhibition of the BMP signaling pathway, hMSGSCs retained their stem cell characteristics but did not differentiate further into the mature cell type, implying that Noggin and CHIR99021 may be important factors to obtain hMSGSCs in vitro*.*

## Conclusions

We found that during in vitro culture, salivary gland stem/progenitor cells were purified and enriched, whereas epithelial progenitor cells were unable to stably maintain their cell state and differentiated. The Wnt and BMP pathways played an important role in regulating the self-organization and differentiation of hMSGSCs. Activation of the WNT/β-catenin signaling pathway and inhibition of the BMP pathway increased the proportion of hMSGSCs and inhibited their growth in an in vitro 3D culture system. Using this research model, we can explore other signals that affect the development of ectoderm organs in future studies. These findings will contribute to the development of a regenerative method for salivary gland organoid generation with functions of hMSGSCs, thereby providing strategies for cell therapy and organoid regeneration.

### Supplementary Information

**Additional file 1. **Product ID of Antibodies and Primer sequences.

**Additional file 2. **Measurement of the diameter of hMSGSCs spheres upon supplementation with different signal pathway factors (day 10).

## Data Availability

The data generated or analyzed during this study are included in this article, or if absent are available from the corresponding author upon reasonable request.

## Abbreviations

AMY1B: Amylase alpha 1B
AQP5: Aquaporin 5
BMP: Bone morphogenetic protein
CK: Cytokeratin
CXCR4: C-X-C chemokine receptor type 4
DAPI: 2-(4-Amidinophenyl)-1H–indole-6-carboxamidine
ERK: Extracellular signal-regulated kinase
GFR: Growth factor reduced
GSK3β: Glycogen synthesis kinase 3β
hMSGSCs: Human minor salivary gland stem/progenitor cells
JNK: C-Jun N-terminal kinase
KRT: Keratin
LAM: Laminin
LGR5: Leucine-rich repeat containing G protein-coupled receptor 5
MAP: Mitogen-activated protein
MTT: 3-(4,5-Dimethylthiazol-2-yl)-2,5-diphenyltetrazolium bromide
MUC5B: Mucin 5B
PBS: Phosphate-buffered saline
SMAD: Suppressor of mothers against decapentaplegic
SOX2: SRY-box transcription factor 2
VIM: Vimentin
Wnt3A: Wnt Family Member 3A
