# Supplementary material for: Maintenance of adult stem cells from human minor salivary glands via the Wnt signaling pathway
Source: Stem Cell Res Ther. 2023 Aug 25;14:220. doi: 10.1186/s13287-023-03445-x (PMC10464143; doi:10.1186/s13287-023-03445-x)
Supplement: Supplementary file 1 — Additional file 1. Product ID of Antibodies and Primer sequences. [file 13287_2023_3445_MOESM1_ESM.docx]

**Supplementary Table 1. Product ID of Antibodies**

| **Immunofluorescence staining antibody** | **Product ID** |
| --- | --- |
| KRT15/ CK15 | Abcam, ab80522 |
| KRT19/ CK19 | Abcam, ab76539 |
| CD31 | Abcam, ab76533 |
| VIM | Abcam, ab92547 |
| KRT 14 | Abcam, ab51054 |
| CXCR4 | Abcam, ab124824 |
| AQP5 | Affinity Biosciences, AF5169 |
| LAM | Abcam, ab11575 |
| hTERT | Abcam, ab111584 |
| **Flow Cytometry Antibody** | **Product ID** |
| CD29 | Biolegend, 303015 |
| CD49f | Biolegend, 313607 |
| CK-kit | BD, 550953 |
| HLA-ABC | Biolegend, 311403 |
| HLA-DR | Biolegend, 307605 |
| CD117 | Biolegend, 313213 |
| SSEA-1 | Biolegend, 125609 |
| CD90 | Biolegend, 328115 |

**Supplementary Table 2. Primer sequences used in this study.**

| **Gene** | **Primer sequences (5’-3’)** |
| --- | --- |
| *LGR5* | F:CATCGCAGCAGTGAACAT |
|  | R:GCCAGAGTAAGCAGGAAA |
| *SOX2* | F:CCATGCAGGTTGACACCGTTG |
|  | R:TCGGCAGACTGATTCAAATAATACAG |
| *KRT5* | F:GAGGAATGCAGACTCAGTGGA |
|  | R:CACTGCTACCTCCGGCAAG |
| *KRT7* | F:ATCGACAAGGTGCGGTTTCT |
|  | R:TCAAAGATGTCTGGGAGGCG |
| *KRT14* | F:GCAGCAGAACCAGGAGTACAA |
|  | R:AGGAGGTCACATCTCTGGATGAC |
| *KRT19* | F:TGAGGAGGAAATCAGTACGCT |
|  | R:CGACCTCCCGGTTCAATTCT |
| *MUC5B* | F:TCAAGTCCGCATCAACACGA |
|  | R:CTGAGTACTTGGACGCTCCG |
| *AMY1B* | F:GAGGGGTTCAGGTCTCTCCA |
|  | R:TGTTCCTGCACTCACAGCAT |
| *AQP5* | F:CCGCTCACTGGGTTTTCTGG |
|  | R:TTTGATGATGGCCACACGCT |
| *GAPDH* | F:GCACCGTCAAGGCTGAGAAC |
|  | R:TGGTGAAGACGCCAGTGGA |
| *KRT15* | F: CCAGTCCGTGCCTTAGAG |
|  | R: AGGAACAGCCGCCATACA |
| *Ki67* | F:ACGCCTGGTTACTATCAAAAGG |
|  | R:CAGACCCATTTACTTGTGTTGGA |

**Supplementary figure 1.**


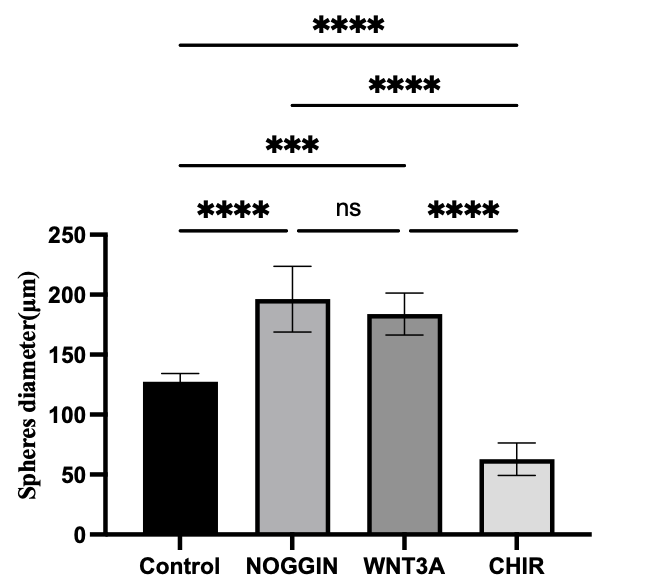


**Supplementary Figure 1. Measurement of the diameter of hMSGSCs spheres upon supplementation with different signal pathway factors (day 10) (*P < 0.05, ****P < 0.0001, ns=non-significant)**
