# Supplementary figures and images for: Maintenance of adult stem cells from human minor salivary glands via the Wnt signaling pathway
Source: Stem Cell Res Ther. 2023 Aug 25;14:220. doi: 10.1186/s13287-023-03445-x (PMC10464143; doi:10.1186/s13287-023-03445-x)

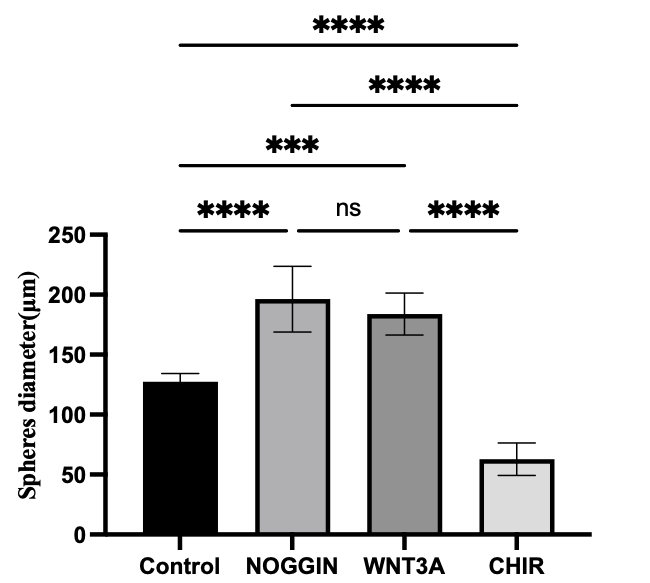

Supplement: Supplementary file 2 — Additional file 2. Measurement of the diameter of hMSGSCs spheres upon supplementation with different signal pathway factors (day 10). [file 13287_2023_3445_MOESM2_ESM.png]
